# Orbital Solitary Fibrous Tumor: Four Case Reports—Clinical and Histopathological Features

**DOI:** 10.1155/2021/5822859

**Published:** 2021-06-09

**Authors:** Carmen Navarro-Perea, Cristina Calleja-García, Álvaro Bengoa-González, María-C. Garrido, Enrique Mencía-Gutiérrez, Silvia Pérez-Trigo

**Affiliations:** ^1^Ophthalmology Department, 12 de Octubre Hospital, Complutense University, 28041 Madrid, Spain; ^2^Pathology Department, 12 de Octubre Hospital, Complutense University, 28041 Madrid, Spain

## Abstract

**Purpose:**

To retrospectively describe the clinical characteristics, management, and outcomes of four cases of orbital solitary fibrous tumor (SFT). In one patient, we present an ultrasonic aspirator system for tumor removal.

**Methods:**

Four patients with orbital SFT were selected: one patient with orbital SFT, another patient with frontal and ethmoidal SFT and orbital affectation with high rates of recurrence, the third patient with frontal lobe SFT and orbital invasion with multiple recurrences, and the fourth case with a history of craniopharyngioma surgery and SFT located on the orbital apex.

**Results:**

All cases showed proptosis, eye movement restriction, and, in three cases, visual acuity alteration. Different treatments were applied: in three cases, excision was performed, one of them with an ultrasonic aspirator system, and in the remaining case, an exenteration was done (in two cases, radiosurgery treatment was also applied). The immunohistochemical study revealed SFT, similar to hemangiopericytomas (HPCs). No recurrence has been observed after surgical treatment.

**Conclusion:**

The SFT is a spectrum of different tumors with similar histopathological characteristics. The use of immunohistochemical markers is very helpful in the diagnosis. The main problem of orbital involvement is the risk of damaging important structures adjacent to the tumor during the surgical removal. The ultrasonic aspirator system allows elimination of the tumor without damaging other orbital structures.

## 1. Introduction

Solitary fibrous tumor (SFT) is a rare tumor which was first described in the visceral pleura in 1931 by Klemperer and Rabin [1]. In the literature, numerous cases are described and there are reviews of extrapleural SFTs; currently, they are thought to appear anywhere in the body [1, 2]. SFT was first described in the orbit in 1994 by Westra et al. [3], and since then, there have been published 150 cases in English literature [4]. They are usually benign, although there are specific malignant cases with frequent local recurrences and invasion of adjacent structures [5, 6]. Stereotactic radiosurgery (SRS) is described in the literature as an adjunctive treatment option when surgical resection is not complete or as a treatment for recurrences [2]. At present, the SFT spectrum includes lesions like hemangiopericytomas (HPCs), giant cell angiofibromas, and fibrous histiocytomas (FHs) [7].

We report four cases of orbital SFT with descriptions of clinical symptoms, immunohistochemical and histopathological details, and treatment. In one case, we also describe the use of SONOPET® (Stryker, Kalamazoo, MI, USA) ultrasonic aspirator system for the tumor excision postincisional biopsy. It is a surgical tool that uses low-frequency ultrasonic vibrations to fragment tissue while simultaneously irrigating and aspirating the surgical field [8].

## 2. Case 1

**Figure 1:**
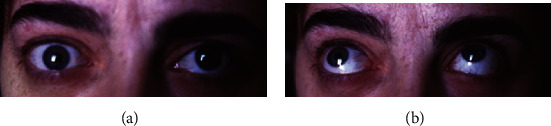
Case 1. (a, b) Clinical photograph shows alteration of the external ocular movements with limitation to supraduction and abduction.

**Figure 2:**
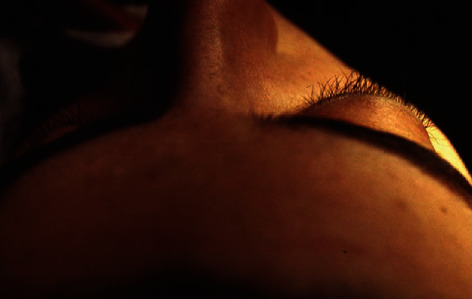
Case 1. Clinical photograph shows proptosis of 9 mm in the right eye.

**Figure 3:**
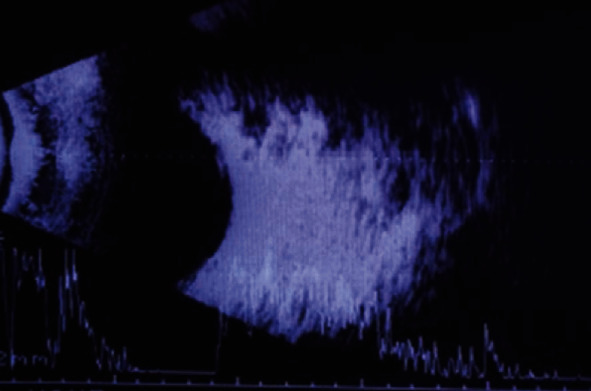
Case 1. Orbital echography shows a superior extraconal mass with high density and irregular pattern.

**Figure 4:**
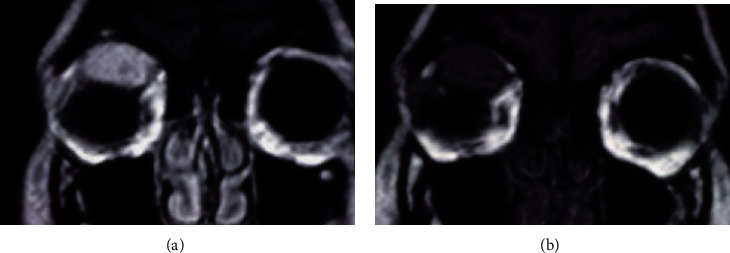
Case 1. (a) Magnetic resonance imaging (MRI) in coronal image showing T2-weighted extraconal tumor based on superior and central orbital, which measures 3.7 cm in the greatest axis and hyperintensity image. (b) T1 weighted with hypointensity image.

**Figure 5:**
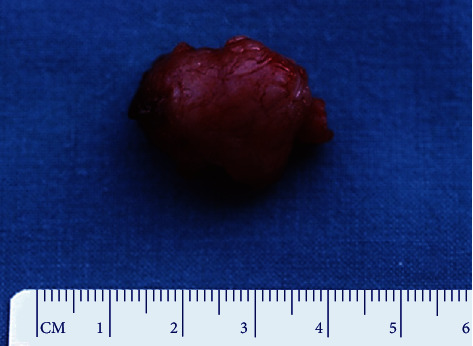
Case 1. Gross examination demonstrated a pink, indurated, well-circumscribed, and encapsulated appearance.

**Figure 6:**
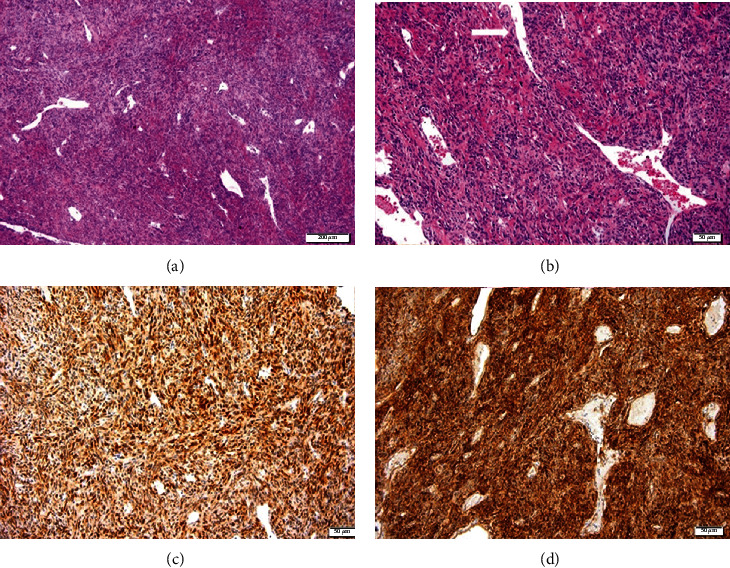
Case 1. (a, b) Microscopic appearance of the tumor, composed of a proliferation of spindle cells arranged in an irregular manner with a focal storiform pattern, bland cytology, and vascular proliferation with a hemangiopericytoma-like growth pattern (arrow in (b)). (H&E, ×40 and ×100 magnification.) Positivity of the tumor for (c) BCL2 and (d) CD34. (Bar is indicated in *μ*m, down and to the right).

The first case is a 25-year-old woman with loss of visual acuity (VA) in the right eye (RE) for one year. The best-corrected visual acuity (BCVA) was 0.5 (decimal scale, Snellen) in the RE and 1.0 in the left eye (LE). In the RE, she also presented alteration of the external ocular movements with limitation to supraduction and abduction (Figures 1(a) and 1(b)), proptosis of 9 mm (Figure 2), lagophthalmos of 3–4 mm, and a mass in the upper orbital region. The LE did not show any alteration. Orbital echography showed an extraconal superior mass with high density and an irregular pattern (Figure 3). In the magnetic resonance imaging (MRI), an extraconal, superior, and central orbital mass was observed; it measured 3.7 × 3.5 × 1.7 cm and extended up to the posterior third of the orbit, with hyperintensity on T2-weighted scans and hypointensity in T1-weighted scans (Figures 4(a) and 4(b)), compatible with cavernous angioma or meningioma. An upper orbitotomy was performed through the upper lid crease with dissection and removal of a compact, depressible pink lesion, which is well defined and without nutrient vascularization (Figure 5). Microscopically, the tumor was composed of a proliferation of spindle cells arranged in an irregular manner with a focal storiform pattern, bland cytology, and vascular proliferation with an HPC-like grown pattern. Immunohistochemistry was positive for CD34, CD99, BCL2, vimentin, and myoglobin (Figures 6(a)–6(d)). The tumor was negative for AE1-AE3 cytokeratin, epithelial membrane antigen (EMA), S100 protein, smooth muscle actin (SMA), and desmin. Mitotic activity showed a low mitosis index (<1–4 high-power fields (HPF)). There was no evidence of hemorrhage, necrosis, or infiltrative borders nor architectural signs of malignancy. After 15 years of follow-up, there has been no evidence of recurrence.

## 3. Case 2

**Figure 7:**
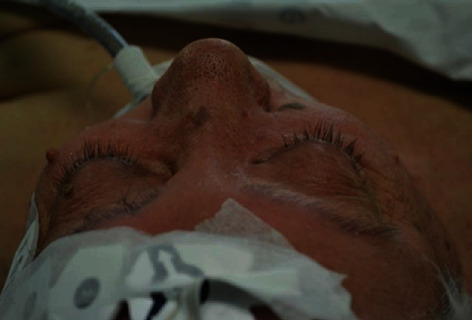
Case 2. Clinical photograph shows proptosis of 4 mm in the right eye.

**Figure 8:**
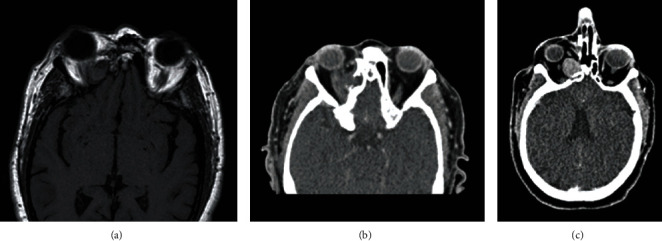
Case 2. (a) MRI in axial view showing the initial large, well-defined round mass over the superior and posterior orbits. Axial computed tomography (CT) imaging showing (b) the third recurrence which includes ethmoidal invasion 3 years later (c) and a fifth recurrence 5 years later.

The second case is a 74-year-old woman who had been receiving follow-up monitoring in otorhinolaryngology for 8 years for a frontal and ethmoidal SFT located on the right side; the SFT had been operated on five times for multiple recurrences despite complete resections. At the last visit, the patient was assessed by ophthalmology because of a 4 mm exophthalmos (Figure 7), with inferior and external displacement of the RE, and limitation to adduction and supraduction. The BCVA was 0.7 (decimal scale, Snellen) in the RE and 0.9 in the LE. Magnetic resonance imaging (Figure 8(a)) and computed tomography (CT) imaging (Figure 8(b)) with contrast showed a 2.2 cm mass that captured contrast; it was located in the right ethmoid sinus with medial invasion into the orbit with infiltration of the medial rectus muscle and minimal posterior extension to the sphenoid (Figure 8(c)). The patient was treated with a lateral orbitotomy to shift the eye, and then, through a transcaruncular approach, the tumor was located. First, samples were taken for histopathological and immunohistochemical study, and then, with the SONOPET® ultrasonic aspirator system, the tumor was completely aspirated.

**Figure 9:**
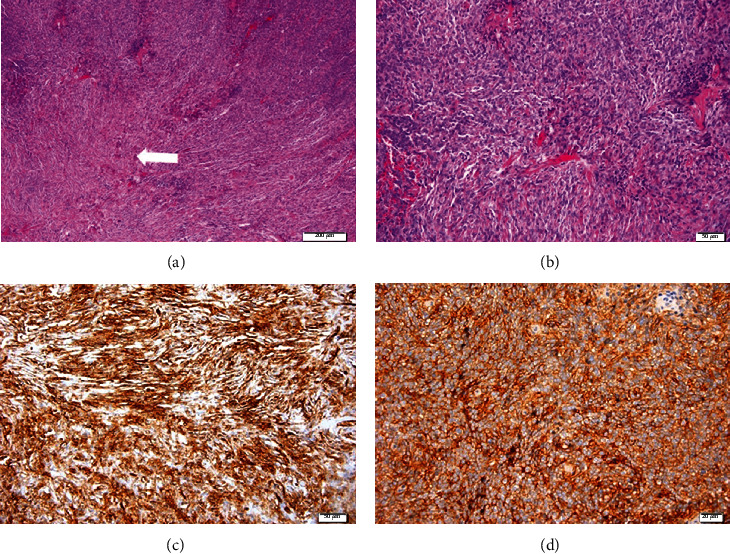
Case 2. (a) Microscopically, the tumor reveals fibroblastic proliferation with hypo- and hypercellular areas alternating with each other and bands of sclerotic collagen fiber networks (arrow points at the more hypocellular area) (H&E, ×40 magnification). (b) Higher magnification demonstrates the cellular phase of the tumor with tightly packed fusiform cells arranged around a ramifying vascular network (H&E, ×100 magnification). Positivity of the tumor for (c) CD34 and (d) CD99. Bar is indicated in *μ*m, down and to the right.

Gross examination revealed a tan-colored tumor. Microscopically, the tumor showed a fibroblastic proliferation with hypo- and hypercellular areas alternating with each other and bands of sclerotic collagen fibers. The arrangement of the cells varied from area to area in the same tumor. In some areas, the cells were arranged in short ill-defined fascicles, with a storiform pattern, whereas in others they were arranged randomly in what is called a “patternless” pattern. In the most cellular phase of the tumor, tightly packed fusiform cells were arranged around a ramifying vascular network. Commonly, the vessels, particularly the large ones, showed a thick coat of collagen. There was no significant cellular atypia and mitosis was 1–4/10 HPF (Figures 9(a) and 9(b)). On immunohistochemistry, the tumor cells had typically strong and diffuse positivity for vimentin and BCL2, focal positivity for CD34, and weak diffuse positivity for CD99 but negativity for cytokeratins AE1–AE3, actin HHF35, actin 1A4, calponin, caldesmon, desmin, S-100 protein, and collagen IV (Figures 9(c) and 9(d)). With these findings, a final diagnosis of SFT was made. Treatment consisted of 35 Gy at a fractionation of 5 weekly sessions of 5 Gy, by 4 fields shaped static at an energy of 6 MV by multilamines. After this treatment, the patient presented great improvement in right supraversion and abduction with significant reduction in size. No recurrence was observed after 4 years of follow-up. The patient died of a stroke.

## 4. Case 3

**Figure 10:**
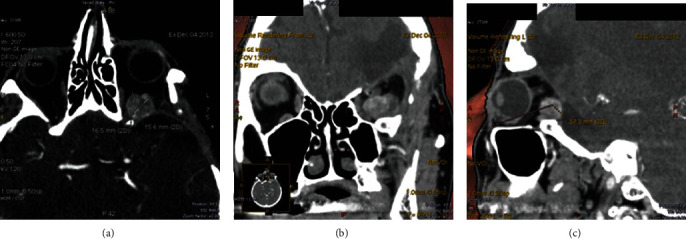
Case 3. (a) CT imaging showing a well-defined rounded lesion, in the left orbital apex. (b) Coronal view. (c) Sagittal view.

**Figure 11:**
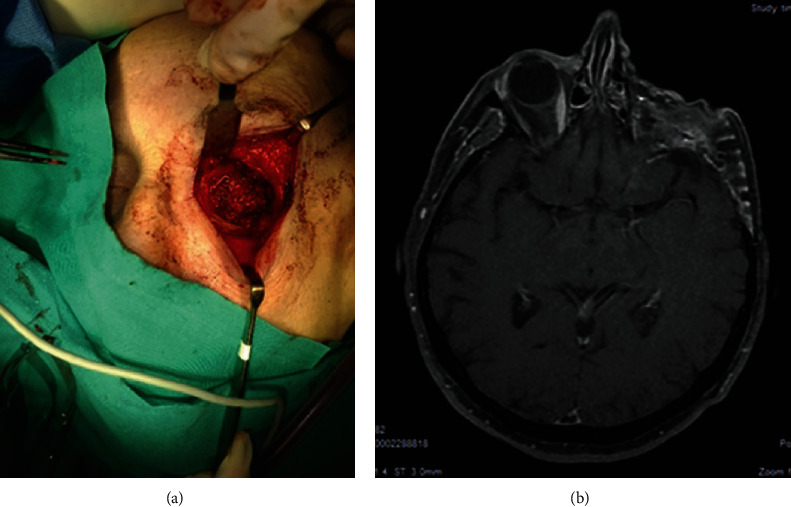
Case 4. (a) Photograph of surgical exenteration. Temporal muscle fills the posterior part of the right orbit. (b) Axial view MRI postexenteration.

The third case is a 78-year-old woman in follow-up by neurosurgery for an SFT at the base of the right frontal lobe which had been operated on eight times for multiple recurrences despite resections. In those operations, part of the lateral and upper wall of the right orbit had been resected due to tumor invasion. The new recurrence was located on the left orbital apex. It was rounded and well defined with a diameter of 16 mm (Figures 10(a)–10(c)). The BCVA in the LE was 0.4 (decimal scale, Snellen), and there was amaurosis in the RE (the eye had been blind for 2 years) with painless exophthalmos and eye movement limitation. Due to the aggressive nature of the tumor, exenteration and reconstruction of the left orbit with a temporal muscle flap were performed (Figures 11(a) and 11(b)). The immunochemical study showed positive markers like vimentin, CD34 (positive focal), BCL2, CD99, S-100 protein, and MIB-1 (low positivity) and negative markers like actin, calponin, AE1–AE3 cytokeratins, caldesmon, desmin, and collagen IV. Cellular atypia and mitosis were >10 HPF. The histopathology confirmed SFT, which is HPC type. After the pathological confirmation and due to the high rate of recurrences of the tumor, SRS was indicated as adjunctive treatment. No recurrence has been observed after 5 years of follow-up.

## 5. Case 4

**Figure 12:**
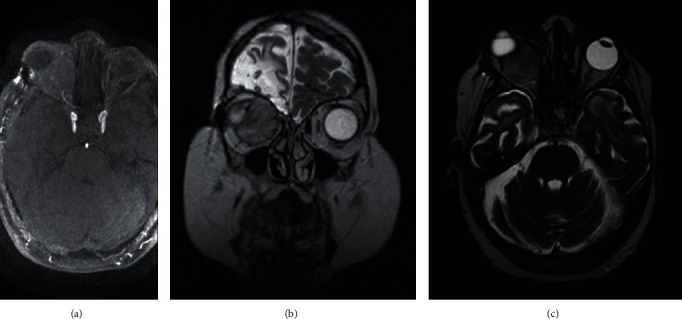
Case 4. (a) CT imaging with a great lesion, hyperdense morphology appears in the right intraconal region. The lesion imprints on the back of the globe, as well as the optic nerve and internal rectus muscle, with close contact with the external rectus muscle. (b, c) MR imaging, coronal view, presents lesion isodensity in T1- and T2-weighted sequences.

**Figure 13:**
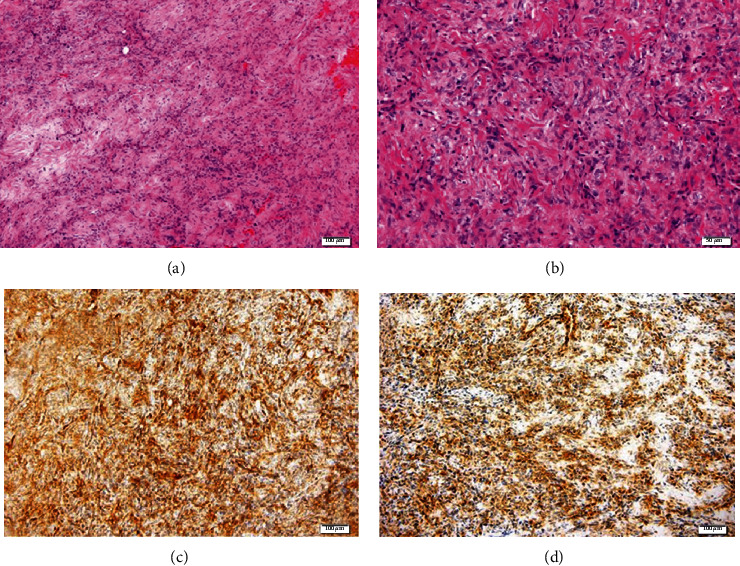
Case 4. (a) It shows the histological appearance of a moderately fibroblastic neoplasia with a mostly storiform pattern. The tumor is composed of bland and uniform oval to spindle cells dispersed along thin parallel collagen bands with hypocellular areas (H&E, ×100 magnification). (b) Hypercellular areas (H&E, ×200 magnification). Positivity for (c) CD34 and (d) BCL2. Bar is indicated in *μ*m, down and to the right.

The fourth case is a 44-year-old woman with a history of craniopharyngioma surgery due to neurosurgery at 9 and 12 years of age. She showed proptosis in the right side, which had begun one week previously. She had amaurosis in the RE and BCVA in the LE 0.8 (decimal scale, Snellen). She had iatrogenic secondary panhypopituitarism due to previous neurosurgery and epilepsy (absence-type seizures with right frontal epileptiform focus). Given the suspicion of a possible recurrence, a CT scan was performed; it showed a lesion of 24 × 24 × 26 mm with a rounded, hyperdense morphology, located in the medial intraconal region of the right orbit. The lesion imprinted on the back of eyeball, as well as the optic nerve and internal rectus muscle, with direct contact with the external rectus muscle. Due to the previous neurosurgery, there was residual frontal cerebral lobe basal hypodensity, as well as hypodensities in white matter of both convexities in the frontal and temporal vertebral lobes (Figure 12(a)). In the MRI, the lesion presented isodensity with the extraocular muscles in T1- and T2-weighted sequences. After the introduction of intravenous contrast, an intense enhancement was observed except for a small hypocaptant focus in its anterior part. There was bone remodeling of the medial orbital wall and involvement of the optic nerve due to contiguity. In the cranial study, areas of signal hyperintensity were evident in T2 and FLAIR sequences in the bilateral frontobasal white substance (Figures 12(b) and 12(c)), bilateral temporobasal substance, and both internal capsules, all related to residual ischemic events/gliosis. A debulking was performed through a lateral orbitotomy. Microscopic study showed a moderate–highly cellular neoplastic proliferation, constituted by cells of mesenchymal-type morphology that were arranged forming bundles. The cells were spindle shaped, with oval nuclei, scarcely pleomorphic and without nucleolus; cytoplasms were eosinophilic and poorly defined. The mitotic index was 1/10 HPS. Some areas of the lesion were less cellular, showing a myxoid stroma, while in other areas the stroma was dense with a collagenous aspect. There were also numerous vascular clefts “in deer antler.” Necrosis or hemorrhage foci were not observed (Figures 13(a) and 13(b)). In the immunohistochemical study, tumor cells showed positivity for CD34, CD99, and BCL2 and negativity for EMA, protein S100, and glial fibrillary acidic protein. There was negativity for CD31 except in the vascular endothelium (Figures 13(c) and 13(d)). A diagnosis for FST, HPC type, was made. No recurrence was observed after 2 years of follow-up. The patient died from infectious pericarditis and pneumonia.

## 6. Discussion

SFT is a rare fusocellular benign tumor that is more common in the visceral pleura [1] but can appear in extrapleural locations including the pericardium, mediastinum, peritoneum, paranasal sinuses, and orbit [9, 10]. After a systematic review, we found that 150 cases of orbital SFT have been described in the English literature [4]. Usually, orbital SFTs exhibit a benign course, but malignant forms with high rates of local recurrence have been reported [1]. The main orbital location is the upper and medial zone of the orbit, as in our four cases, but they have been described in many other locations [11].

This tumor appears mainly in the fifth decade of life, but it can appear any time from age 15 to over 89 [8]. All of our cases were adult patients (range: 25 to 78 years, mean: 55). In our study, all cases of SFT were in female patients, but the literature shows that there is no gender-based predilection [8, 12]. The most common form of clinical presentation is with unilateral painless proptosis, with slow growth; it may be associated with eyelid edema, visual alterations, the presence of a palpable mass, tearing, and ipsilateral ptosis [1, 11]. Our four cases showed unilateral painless exophthalmos and eye movement limitation. Three patients also showed loss of VA from the beginning, although the literature reveals that SFT does not often cause compression of the optic nerve [4].

In the radiological exam, SFT may be difficult to distinguish from other orbital lesions such as cavernous hemangioma, schwannoma, localized neurofibroma, varix, and orbital metastasis [4]. The computed tomography and MRI usually show a solitary well-defined soft tissue mass [4, 13] that can be located anywhere on the orbit but is more frequent in superior quadrants [4]. The pattern of contrast enhancement and washout on dynamic imaging may help to differentiate between cavernous hemangioma, SFT, and schwannoma [4]. Bone remodeling is present in cases of suspicious malignant transformation, but this is uncommon [13]. In our study, two cases show bone remodeling and high rates of recurrences, leading to suspicion of malignancy. The ultrasound shows a tumor that is well circumscribed, firm, or minimally compressible, variably vascular, with a regular internal structure and low to medium reflectivity [4].

The classic histopathological features of SFT are cells which are described as spindle shaped with a small cytoplasm and indistinct nucleoli, and the tumor matrix contains a distinctive thick “ropy” type of collagen between tumor cells [8, 14]. SFT should be differentiated from other spindle-shaped cell orbital tumors, such as fibrous histiocytoma, HPC, meningioma, and schwannoma [14]. The use of immunohistochemical markers is very helpful in diagnosing SFT. In the literature, there is great controversy about the diagnosis of SFT and HPC since they have similar morphological and immunohistochemical characteristics and there has been a great deal of discussion regarding whether or not they are the same clinical entity.

SFTs show strong and diffuse positivity with CD34 (90–100%) [8–10, 13], vimentin, BCL2, and CD99 [15, 16] and negativity with keratin, cytokeratin, EMA, S100, SMA, and desmin [14]. The CD34 protein is a transmembrane protein that is expressed in vascular and hematopoietic tissue. Smooth muscle tumors such as leiomyoma and leiomyosarcomas show strong positivity for actin and desmin and negativity for S100, BCL2, and CD34. Neural tumors such as schwannoma and neurofibroma can show focal positivity for BCL2 and CD34 and strong positivity for S100 protein. SFT, FH, and HPC all show positive reactivity with vimentin, BCL2, and CD34 [14]. A new classification, published in 2015, includes them in the same entity [3, 17]. Although they are currently included in the same entity, several subtypes of SFT have been differentiated, FH, HPCs, and another lipomatous type [18]. The HPC is originated in the pericytes of blood vessels and usually shows a rich vascular component. HPC cells show high reactivity against vimentin and CD34 but lack immunoreactivity for EMA [9]. FH shows that histiocytes are positive for CD68 but show some variation in CD34 reactivity [9]. CD34 is fairly sensitive for SFT, but it is relatively nonspecific [4]. Recently, NAB2-signal transducer and activator of transcription 6 (STAT6) gene inversion at 12q13 may be identified with an immunohistochemical antibody and it has high sensitivity and specificity for SFT [4, 19, 20].

SFT is a tumor classified as benign, but it can exhibit aggressive behavior with invasion of adjacent and distant tissues and the potential to become malignant. In these cases, high cellularity, mitotic activity > 4/10 HPF (high-power fields), nuclear pleomorphism, necrosis, and large tumors are usually found, although cases in which they did not show these characteristics but have recurrences are also described [10, 11].

For this reason, the treatment of choice for SFT, HPC, and FH is a complete excision of the tumor and careful follow-up in order to prevent recurrences and progression. In HPC, the histopathological features do not always correlate with biological behavior, including malignant potential [9, 21]. Our first case was treated with complete surgical excision of the tumor. In the second case, we used the SONOPET® ultrasonic aspirator system. This is a surgical tool that uses low-frequency ultrasonic vibrations to fragment tissue for tumor excision while simultaneously irrigating and aspirating the surgical field [8]. This device works by ultrasound and has been used previously in brain tumors. It is the first case that we have found in the literature in which it has been used for ophthalmological surgery. It proved to be very useful because it allowed a complete excision of the tumor reaching the ethmoid bone, without damaging adjacent structures. This was possible due to the higher water content and lower collagen and elastin content of the tumor tissues, compared to that of healthy tissues. Because SONOPET® destroys and aspirates the tumor, samples must be taken before the surgery so that a pathological study can be performed. In this case, although the tumor was completely removed, we treated this patient with SRS because of the previous high rate of recurrence.

The third case was treated with exenteration because of the high rate of recurrence and the great orbital affectation; SRS treatment was also implemented. All cases were carefully monitored with no recurrences during this time of follow-up.

The fourth case is a patient with a history of craniopharyngioma surgery in pediatric age; the tumor was resected due to a possible recurrence [22].

## 7. Conclusion

Orbital SFT is a rare benign tumor with a high rate of recurrence and sometimes with malignancy. Orbital location hinders complete surgical removal of the tumor, and this increases the risk of recurrence. By using the SONOPET® ultrasonic aspirator system, it is possible to perform a more complete removal of the tumor without damaging orbital structures adjacent to the tumor, thus increasing surgical success and patient survival.

## Data Availability

The data used to support the findings of this study are available from the corresponding author upon reasonable request.
